# Interplay between colorectal cancer-related lifestyles and the gut microbiome: an exploratory analysis of metagenomic data

**DOI:** 10.1007/s10552-026-02144-1

**Published:** 2026-03-11

**Authors:** Rilla Tammi, Mirkka Maukonen, Niina E. Kaartinen, Kari Koponen, Teemu Niiranen, Guillaume Méric, Demetrius Albanes, Johan G. Eriksson, Pekka Jousilahti, Seppo Koskinen, Anne-Maria Pajari, Rob Knight, Aki S. Havulinna, Veikko Salomaa, Satu Männistö

**Affiliations:** 1 Department of Public Health, Finnish Institute for Health and Welfare (THL), Helsinki, Finland; 2 Department of Public Health, Faculty of Medicine, University of Helsinki, Helsinki, Finland; 3 Folkhälsan Research Center, Helsinki, Finland; 4 Department of Bacteriology and Immunology, University of Helsinki, Helsinki, Finland; 5 Institute of Dentistry, University of Eastern Finland, Kuopio, Finland; 6 Department of Internal Medicine, University of Turku, Turku, Finland; 7 Division of Medicine, Turku University Hospital, Turku, Finland; 8 Systems Medicine & Population Health Division, Department of Life Sciences, University of Bath, Claverton Down, Bath, UK; 9 Cambridge Baker Systems Genomics Initiative, Baker Heart and Diabetes Institute, Melbourne, Australia; 10 Department of Cardiovascular Research, Translation, and Implementation, La Trobe University, Melbourne, VIC Australia; 11 Department of Cardiometabolic Health, University of Melbourne, Melbourne, VIC Australia; 12 Division of Cancer Epidemiology and Genetics, National Cancer Institute, National Institutes of Health (NIH), Bethesda, MD USA; 13 Department of General Practice and Primary Health Care, University of Helsinki and Helsinki University Hospital, Helsinki, Finland; 14 Department of Food and Nutrition, University of Helsinki, Helsinki, Finland; 15 University of California San Diego, La Jolla, CA USA; 16 Institute for Molecular Medicine Finland, FIMM-HiLIFE, Helsinki, Finland; 17 Department of Computing, University of Turku, Turku, Finland; 18 Finnish Institute for Health and Welfare (THL), P.O. Box 30, 00271 Helsinki, Finland

**Keywords:** Diet, Epidemiology, Lifestyle index, Obesity, Physical activity, Prevention

## Abstract

**Purpose:**

The gut microbiome may modify the associations between lifestyle factors and colorectal cancer (CRC) risk, but their complex interplay, including the interactions between lifestyle factors, remain underexplored. We examined associations between CRC-related lifestyle patterns and gut microbiome diversity and composition in Finnish adults.

**Methods:**

Our data included 1,228 adults aged 25–64 years from the National FINRISK/FINDIET 2002 Study. Information on lifestyle and background factors was obtained through self-administered questionnaires. Dietary data were gathered using a 48-h dietary recall. CRC-related lifestyles were modelled using a CRC lifestyle index based on nine major risk factors for CRC. Lower index points reflected higher-risk lifestyles. The gut microbiome profiles were analyzed using shallow shotgun metagenome sequencing. Associations between the index and microbial diversity and composition were assessed using, e.g., linear regression and permutational multivariate ANOVA adjusted for relevant confounders.

**Results:**

The index explained 0.2% of the variation in microbial composition between participants (*p* < 0.05). Higher-risk lifestyles for CRC were associated with lower microbial diversity (*β* 0.037, *p* 0.009). Higher-risk lifestyles were also associated with a higher relative abundance of species representing primarily the family Lachnospiraceae and genera such as *Dorea* and *Mediterraneibacter*, and lower relative abundance of species within the genus *Bifidobacterium* (< 0.0001).

**Conclusions:**

Participants with higher- and lower-risk lifestyles showed clear differences in their gut microbiome diversity and composition, higher-risk lifestyles being associated with potentially adverse microbial traits. These findings contribute to identifying microbial features that may characterize early stages of CRC development in individuals with high-risk lifestyles.

**Supplementary Information:**

The online version contains supplementary material available at 10.1007/s10552-026-02144-1.

## Introduction

Colorectal cancer (CRC) is one of the most common cancer types and leading causes of cancer mortality worldwide [1]. Its global incidence has increased substantially over recent decades and continues to rise, particularly in younger age groups.

Epidemiological findings indicate that lifestyle and anthropometric factors contribute significantly to CRC development; however, the biological mechanisms underlying these associations remain unclear [2, 3]. At the same time, disturbances in the gut microbiome composition and function have been linked to CRC pathogenesis, suggesting a potential pathway between the risk factors and disease onset [4, 5]. Indeed, several major risk factors for CRC, such as obesity, physical inactivity, and high consumption of red and processed meat, have been associated with potentially carcinogenic changes in the gut microbiome [5].

While prior research has identified associations between individual CRC risk factors and the gut microbiome, combinations of risk factors, and their potential interactions, have been less explored. Observational studies have suggested that combinations of certain risk factors increase CRC risk more than individual factors alone, with some evidence of synergy [6–9]. For example, in a study of 170,000 Finnish adults, pairwise combinations of major risk factors, such as alcohol consumption and overweight, were associated with a higher CRC risk compared with individual factors [6]. Alcohol and smoking, in particular, were jointly associated with a higher CRC risk in women than anticipated based on their individual associations. As the risk factors tend to cluster and may interact in complex ways, their combined associations with the gut microbiome need to be explored. Furthermore, in population-based studies, only a small proportion of microbiome variation has been attributed to individual lifestyle factors such as diet, whereas the influence of external factors combined appears to surpass that of genetic factors [10–12]. Consequently, considering combinations of risk factors may provide a more comprehensive perspective on the interplay between lifestyles, the gut microbiome, and CRC risk.

In this study, we aimed to account for the complex interplay between lifestyle and anthropometric factors and the gut microbiome in the context of CRC risk. To achieve this, we studied the associations between CRC-related lifestyles and gut microbiome diversity and composition.

## Methods

### Participants

**Fig. 1:**
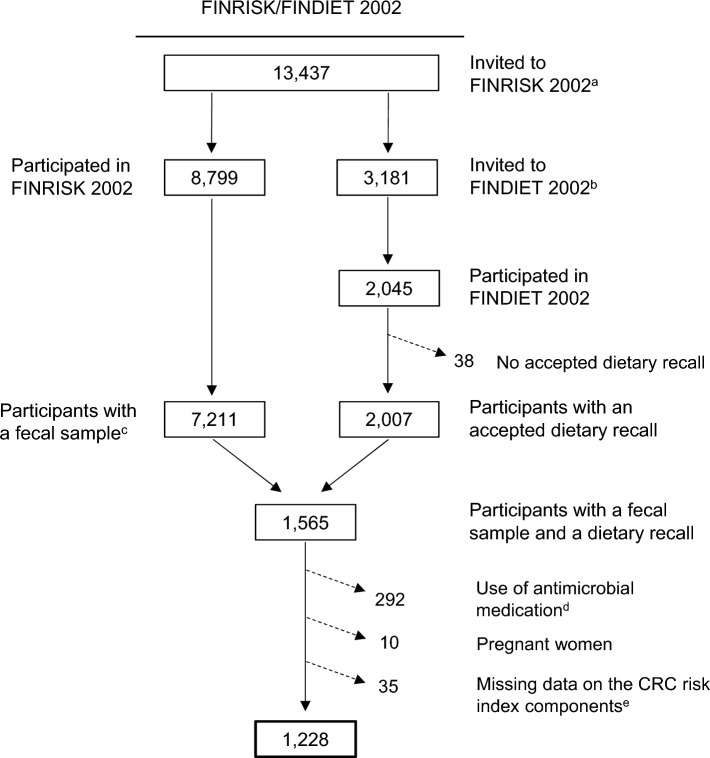
Exclusions and the final study sample in FINRISK/FINDIET 2002. ^a^A random sample of adults aged 25 − 74 years from six large geographical areas of mainland Finland. ^b^The FINDIET subsample was randomly selected from individuals invited to FINRISK 2002. ^c^Successfully sequenced fecal sample with sufficient read count (≥ 50,000). ^d^Registered purchase of systemic antibacterial medication (Anatomical Therapeutic Chemical classification code J01) within 6 months prior to the baseline examination. ^e^Missing data on waist circumference *n* = 1, physical activity *n* = 6, and alcohol intake *n* = 28

Our data comprised participants from the National FINRISK/FINDIET 2002 Study (FINRISK/FINDIET 2002) [13]. FINRISK 2002 is part of the FINRISK study series conducted every 5 years from 1972 to 2012 by the Finnish Institute for Health and Welfare (THL) to monitor health behavior and risk factors of chronic diseases in Finnish adults. In these studies, data were collected in a health examination and through self-administered questionnaires. In FINRISK 2002, participants were also asked to donate a fecal sample. Additionally, FINRISK 2002 included a sub-study called FINDIET 2002, in which diet was assessed using a 48-hour dietary recall (48-h recall) [14]. In the current study, we included participants with a fecal sample and an accepted dietary recall (Fig. 1). We excluded participants with a registered purchase of systemic antibacterial medication [Anatomical Therapeutic Chemical (ATC) Classification Code J01] within the 6 months preceding the baseline examination, pregnant women, and those with missing information on the risk factors included in the CRC lifestyle index. The final study population included 1,228 participants aged 25–74 years.

Additionally, we used pooled data from five large cohorts of Finnish adults to test the performance of the CRC lifestyle index in relation to CRC risk, prior to other analyses. These cohorts included the Alpha-Tocopherol Beta-Carotene Cancer Prevention Study (ATBC) [15], the Health 2000 Health Examination Survey (Health 2000) [16], the Helsinki Birth Cohort Study (HBCS) [17], the DIetary, Lifestyle and Genetic Determinants of Obesity and Metabolic Syndrome 2007 Study (DILGOM 2007) [18], and the National FINRISK 2012 Study (FINRISK 2012) [13] (Online Resource 1). In each cohort, data were collected through a health examination and self-administered questionnaires, including a food frequency questionnaire (FFQ). The cohorts were followed using national health registers. The final study sample included 43,488 participants aged 25–99 years (Online Resource 2). The methods described below apply to FINRISK/FINDIET 2002 and the pooled cohorts unless otherwise indicated.

### CRC lifestyle index

To model CRC-related lifestyles, we adapted the standardized 2018 World Cancer Research Fund/American Institute for Cancer Research (WCRF/AICR) Score [19] to include only CRC-specific risk factors. The standardized score operationalizes the general cancer prevention recommendations of the 2018 WCRF/AICR Third Expert Report [20]. Based on the same report, we included in the index nine lifestyle and anthropometric factors with strong evidence of causality for CRC. These were body mass index (BMI), waist circumference (WC), height, leisure-time physical activity, consumption of whole grains, dairy products, and red and processed meat, and intake of alcohol (Table 1). Each component was assigned 0, 0.5, or 1 point representing not meeting, partially meeting, or meeting the target cut-off [19]. The cut-offs were determined based on the literature, primarily the 2018 WCRF/AICR Report (Table 1) [20]. As exceptions, height was scored using sex-specific tertiles because cut-offs could not be determined, and physical activity was scored based on predefined categories of the variable available in our data.

**Table 1:** The components and scoring of the CRC lifestyle index

	Scoring		
Component	0	0.5	1
Body fatness^a^			
BMI^b^ (kg/m^2^)	≥ 30	25– < 30	< 25
WC^b^ (cm)			
Women	≥ 88	80–< 88	< 80
Men	≥ 102	94–< 102	< 94
Height^c^ (cm)	Tertile 3	Tertile 2	Tertile 1
Leisure-time physical activity^d^	Inactive	Moderately active	Active
Diet^e^			
Whole grains^f,g^ (g/day)	< 45	45–< 90	≥ 90
Dairy products^g^ (g/day)	< 200	200–< 400	≥ 400
Red and processed meat^h^ (g/week)	Red meat > 350 or processed meat ≥ 100	Red meat ≤ 350 and processed meat 21 − < 100	Red meat ≤ 350 and processed meat ≤ 21
Alcohol (100%)^i^ (g/day)	> 20	> 0 − ≤ 20	0

*BMI* body mass index, *WC* waist circumference

^a^The points for BMI and WC were averaged to calculate one score for body fatness. Body fatness was based on BMI alone if data on WC was missing in the entire dataset

^b^The cut-offs are based on the 2018 WCRF/AICR Third Expert Report [20] and the WHO guidelines[27, 28]

^c^Height was scored based on sex-specific tertiles. Tertile medians (cm) in FINRISK/FINDIET 2002 were in women T1: 157, T2: 163, T3: 169; in men T1: 170, T2: 176, T3: 183

^d^Leisure-time physical activity was scored based on predefined categories of the variable available in our data: inactive (only light activities, e.g., reading), moderately active (e.g., walking ≥ 4 h/week), or active (e.g., running or competitive sports ≥ 3 h/week)

^e^The points for the consumption of whole grains, dairy products, and red and processed meat, and intake of alcohol were averaged to calculate one dietary score

^f^Whole grain consumption was calculated as the sum of rye, oat and barley consumption[29]

^g^The cut-off for meeting the target consumption was based on the 2018 WCRF/AICR Third Expert Report [20]. The cut-off for partially meeting the target consumption was defined as consuming at least half of the target amount[19]

^h^The cut-offs are based on the 2018 WCRF/AICR Third Expert Report [20], the standardized 2018 WCRF/AICR Score [19] and the Nordic Nutrition Recommendations 2023[30]

^i^The cut-offs are based on the 2018 WCRF/AICR Third Expert Report[20]

As per the standardized score, we averaged the points for BMI and WC to form one body-fatness score. In one of the pooled cohorts (ATBC), WC was not measured, for which the score was based on BMI alone [19]. To avoid overemphasizing diet, we also averaged the points for the four dietary components to form a single dietary score. Finally, the points for body fatness, height, physical activity, and diet were summed, resulting in scores ranging between zero and four points, with higher points indicating fewer CRC risk factors (i.e., lower-risk lifestyles for CRC).

From the strong-evidence risk factors identified in the 2018 WCRF/AICR Report [20], we omitted dietary fiber to avoid redundancy with whole grains. Whole grains were prioritized because the evidence of associations with a reduced CRC risk has been strongest for cereal fiber [21, 22], and whole grains are a major source of both cereal fiber and total fiber in Finland [23]. Moreover, the protective effects of whole grains may extend beyond the role of fiber per se, owing to their various potentially beneficial bioactive compounds [24, 25]. We also omitted the use of calcium supplements because of the high dairy product consumption in Finland, for which dietary calcium intake is generally sufficient in adults [23, 26].

### Dietary factors

In FINRISK/FINDIET 2002, detailed dietary information was collected using a 48-h recall capturing food consumption over the two preceding days [26]. Trained nutritionists interviewed participants during the health examination, documenting all foods and beverages consumed within the study period. Portion sizes were estimated based on a validated portion size picture booklet [31], household measures, and standard food packaging. To estimate alcohol intake, we used data collected through self-administered questionnaires to better capture habitual use. The data included the number of portions and frequency of consumption of different alcoholic beverages over the past year.

In the pooled cohorts, dietary information was collected through validated semi-quantitative FFQs that assessed habitual food consumption over the past year [32 – 35]. The FFQ included approximately 130 food items and mixed dishes (276 in ATBC), and the consumption of each item was recorded by up to nine frequency categories and fixed portion sizes.

The average daily food consumption (g/day) and energy intake (kJ/day) were calculated using the Finnish Food Composition Database Fineli® and an in-house software [36]. Weekly consumption was calculated by multiplying daily consumption by seven. Food consumption was calculated at the ingredient level by breaking down foods and mixed dishes into basic ingredients using Fineli® standard recipes. Whole grain consumption was calculated as the sum of rye, oat, and barley consumption, which has been shown to correlate strongly with total whole grain intake in Finnish adults (*r* = 0.99) [29]. Dairy products included milk, fermented dairy products (e.g., yoghurt), cheese, cream, and ice cream. The contribution of ice cream on dairy consumption was small. Red meat included beef, pork, and lamb. Processed meat included sausages and cold cuts.

### Physical activity and anthropometric factors

Information on leisure-time physical activity was collected through self-administered questionnaires. Based on the collected data, participants were categorized into inactive (only light activities, e.g., reading), moderately active (e.g., walking ≥ 4 h/week), and active (e.g., running and competitive sports ≥ 3 h/week) [37].

Weight (kg), height (m), and WC (cm), were measured in the health examination by trained research staff using international standard protocols [38]. BMI was calculated as weight divided by height squared (kg/m^2^).

### Confounding factors

Information on participants’ sex and age originated from the sampling frame (Finnish Population Information System). Information on educational attainment, smoking history, and use of hormone-replacement therapy (HRT) were collected through self-administered questionnaires. Educational attainment was categorized into low, middle, or high. Smoking status was categorized into current, former, or never smoker. In ATBC, all participants were male and current smokers at baseline owing to the study design. In FINRISK/FINDIET 2002, current smokers were combined with former smokers who had smoked within the past 6 months (‘current smokers’) and never smokers with those who had not smoked within the past 6 months (‘nonsmokers’) to create a binary variable. HRT use (in women) was categorized as ‘ever’ or ‘never’. Educational attainment and HRT use were adjusted for only in testing the CRC index.

Information on the use of potentially microbiome-altering medication was obtained from the prescription medicine purchase register maintained by the Social Insurance Institution of Finland. In addition to systemic antibacterial medication, we considered metformin (ATC code A10BA02), psycholeptics (NO5), psychoanaleptics (NO6), proton pump inhibitors (A02BC), and constipation medication (A06A).

Information on baseline diabetes status was obtained from the nationwide administrative registries on reimbursement for diabetes medication expenses and medication purchases (ATC codes A10), hospitalizations, or causes of death (International Classification of Diseases 10 (ICD-10) codes E10–14) [39]. Participants were linked to the registries using unique personal identity codes issued to all Finnish citizens and permanent residents. Diabetes status was adjusted for in testing the CRC index, but not in the microbiome analyses due to overlap with metformin use.

### Colorectal cancer ascertainment

Data on CRC diagnoses (ICD-9 codes 153, 154.0, 154.1 or ICD-10 codes C18, C19.9 and C20.9) were obtained from the Finnish Cancer Registry [40]. Participants were linked to the registry using the unique personal identity codes. The follow-up extended from the date of enrolment until CRC diagnosis, death, or the end of study period (see Online Resource 1 for the pooled cohorts).

The final FINRISK/FINDIET 2002 study sample did not include participants with prevalent CRC at baseline. During the follow-up (until 31 December 2022), 12 participants were diagnosed with CRC. Of these, all but one were diagnosed more than 8 years after the baseline.

### Microbiome characterization

All participants in FINRISK/FINDIET 2002 willing to donate a fecal sample received a sampling kit during the health examination with detailed instruction on its use. The participants were instructed to gather the sample at home at their earliest opportunity and mail it overnight to the THL Laboratory. The samples were collected into 50-ml Falcon tubes without a stabilizing solution and mailed under typical Finnish winter conditions. Upon receipt, the samples were frozen and stored at − 20 °C until sequencing in 2017 at the University of California San Diego.

A detailed description of the DNA extraction and library preparation has been published elsewhere [41]. The samples were characterized using shallow shotgun whole-metagenome sequencing with the Illumina Hi-Seq 4000 instrument (Illumina, Inc.), generating 150-bp paired-end reads [42]. The mean read count per sample was approximately 900,000. Adapter sequences were removed using fastp (version 0.23.4) [43]. Host sequences were filtered by mapping reads against the human pangenome, T2T-CHM13v2.0, and GRCh38 using minimap2, followed by extraction of unmapped reads with samtools [44]. Raw sequences were then taxonomically annotated using incorporated metagenomes in Greengenes2 [45].

We measured alpha diversity, which quantifies within-individual microbial diversity, using the Shannon index based on species-level raw counts [46]. Beta diversity, which quantifies between-individual variation in microbial community composition, was measured using the weighted UniFrac metric based on relative abundances [47]. Relative abundances were calculated from the annotated data by scaling the number of raw read counts of each taxon to the total sum of reads. Differential abundance analyses were conducted using species-level raw counts, excluding rare species with a prevalence < 1% or a relative abundance < 0.01% [41].

### Statistical analyses

Throughout the study, continuous variables were characterized using medians and interquartile ranges (IQR) and categorical variables using proportions (%). We tested the CRC lifestyle index in relation to CRC risk in the pooled cohorts using two-stage meta-analysis. The index was divided into quintiles cohort-specifically. First, we calculated cohort-specific hazard ratios (HR) and 95% confidence intervals (95% CI) using multivariate Cox regression models [48]. The estimates were subsequently pooled using random-effects models, with weights equal to the inverse of their variances. The proportional hazards assumption was met. *p* for trend over the index quintiles was calculated using the quintile medians as continuous independent variables. Heterogeneity between the cohorts was tested using *Q*-statistics. The cohort-specific analyses were adjusted for sex, age, energy intake, educational attainment, and smoking status. Additionally, three sensitivity analyses were conducted: (1) excluding participants diagnosed with CRC within the first 2 years of follow-up, (2) further adjusting for prevalent diabetes, and (3) further adjusting for HRT use in women. We examined associations between the index components and CRC risk using the same methods.

In the microbiome analyses, we used multiple linear regression to examine associations of the continuous CRC lifestyle index and its components with alpha diversity. To analyze associations between the index and beta diversity, we used Permutational Multivariate Analysis of Variance (PERMANOVA) [49], dispersion analysis, Distance-based Redundancy Analysis (dbRDA) [50], and Principal Coordinates Analysis (PCoA). PERMANOVA was used to test the degree to which the CRC index (and its components) explain the compositional variation in microbiome between individuals [49]. Dispersion analysis was used to test whether differences in beta diversity across the index were driven by within-group variability. DbRDA was used to examine constrained variance, i.e., how much the index and confounders together explain of the variation in beta diversity, and visualize the direction of associations between them. PCoA was used to visualize the clustering of microbial communities in the highest and lowest index quintile, and the function ‘factorfit’ from the package *vegan* to assess the significance of the clustering. PERMANOVA, dbRDA and factorfit were run with 999 permutations.

To study associations between the CRC lifestyle index and all taxa at species level, we used Analysis of Compositions of Microbiomes with Bias Correction 2 (ANCOM-BC2) [51]. As a secondary analysis to further examine species co-occurrence, we clustered the species that were significantly associated with the index and passed the ANCOM-BC2 robustness screening, using Ward's minimum variance method based on proportionality. The optimal number of clusters was determined using Kelley–Gardner–Sutcliffe penalty function [52]. The results were visualized with a heatmap.

The microbiome analyses were adjusted for sex, age, energy intake, smoking status, use of potentially microbiome-altering medication, and BMI (with predictors other than the index, BMI, or WC). Interaction between the index and sex was tested by including an interaction term in the analyses. In the linear regression analyses (alpha diversity) and PERMANOVA (beta diversity), *p* values were corrected for multiple testing using the Benjamini–Hochberg false discovery rate (FDR) procedure [53]. In the differential abundance analyses, multiple testing was controlled internally by ANCOM-BC2 [51]. A two-sided (FDR-corrected) *p* value of < 0.05 was used to indicate statistical significance.

All analyses were conducted and figures generated using R statistical software versions 4.3 and 4.4 [54]. Key packages included *meta* (8.0.1), *mia* (1.12.0), *vegan* (2.6.8), and *microbiome* (1.28.0) [55–58]*.*

## Results

### Testing the CRC lifestyle index

In the pooled cohorts, 79% of the participants were men, and the median follow-up was 14 years (range 7.8–17.9 years), during which 1,118 CRC cases were diagnosed (Online Resource 1). The median age ranged from 50 to 60 years (Online Resource 3). The median points for the CRC lifestyle index ranged from 1.75 to 2.00.

Participants with the highest points in the CRC lifestyle index (Q5) had a 31% lower CRC risk compared with those with the lowest points (Q1, HR 0.69, 95% CI 0.58–0.83; Table 2). Each one-point increase in the index was associated with a 19% decrease in CRC risk (0.81, 0.74–0.88). A one-point increase corresponds to, for example, the difference between being physically active vs. inactive or having both BMI and WC in the lowest vs. highest category. There was no statistically significant heterogeneity between the cohorts. Furthermore, we observed no significant interaction by sex, and the results were similar in men and women. The results remained similar also in the sensitivity analyses (data not shown). Of the individual index components, BMI (1.13, 1.05–1.23; per 5 kg/m^2^), red meat consumption (1.17, 1.00–1.38; per 100 g/day), and alcohol intake (1.03, 1.01–1.04; per 5 g/day) were significantly associated with CRC risk. Overall, these results suggest that the CRC lifestyle index is an appropriate method to distinguish between participants with higher- and lower-risk lifestyles for CRC in Finnish adults.

**Table 2:** Associations (HR and 95% CI) between the CRC lifestyle index (in quintiles and continuously) and CRC risk in the pooled cohorts

	Index quintiles^a^			*p*_trend_^b^	*p*_het._^c^	Continuous index^d^	*p*
	Q1	Q3	Q5				
Number of participants	8,271	8,579	9,511			43,488	
Number of CRC cases	251	196	234			1,118	
HR (95% CI)^e^	1.00	0.71 (0.59–0.85)	0.69 (0.58–0.83)	< 0.0001	0.44	0.81 (0.74–0.88)	< 0.0001

The pooled cohorts included the Alpha-Tocopherol Beta-Carotene Cancer Prevention Study (ATBC), the Health 2000 Health Examination Survey (Health 2000), the Helsinki Birth Cohort Study (HBCS), the DIetary, Lifestyle and Genetic Determinants of Obesity and Metabolic Syndrome 2007 Study (DILGOM 2007), and the National FINRISK 2012 Study (FINRISK 2012)

*CRC* colorectal cancer, *HR* hazard ratio, *95% CI* 95% confidence intervals

^a^Median index points in the CRC lifestyle index quintiles in *ATBC* Q1: 1.000, Q3: 1.875, Q5: 2.625, in *Health 2000* Q1: 1.000, Q3: 1.875, Q5: 2.750, in *HBCS* Q1: 0.875, Q3: 1.750, Q5: 2.750, in *DILGOM 2007* Q1: 1.125, Q3: 2.125, Q5: 3.000, in *FINRISK 2012* Q1: 1.125, Q3: 2.000, Q5: 3.000

^b^*p* for trend was calculated using index quintile medians as continuous independent variables

^c^*p* for heterogeneity between the pooled cohorts was calculated by *Q*-statistics

^d^HR (95% CI) per one-point increase in the index

^e^Adjusted for sex, age (continuous), energy intake (continuous), educational attainment (low/middle/high), and smoking status (never/former/current smoker)

### The CRC lifestyle index and gut microbiome diversity and composition

In FINRISK/FINDIET 2002, 46% of the participants were men and the median age was 48 years (Table 3). The median score for the CRC lifestyle index was 2.0 points, ranging from 1.1 in Q1 to 3.0 in Q5. No participant scored full points. Participants with higher index points tended to have a higher energy intake and were less often current smokers or users of potentially microbiome-altering medication than those with lower points.

**Table 3:** Participant characteristics in FINRISK/FINDIET 2002 (medians [IQR] or proportions [%])

	Total *n* = 1,228	CRC lifestyle index quintiles				
		Q1 *n* = 252	Q2 *n* = 285	Q3 *n* = 248	Q4 *n* = 234	Q5 *n* = 209
Background factors						
Men (%)	46	48	42	42	50	47
Age (years)	48 (18)	49 (17)	47 (17)	47 (18)	49 (19)	47 (21)
Energy intake (MJ/day)	7.6 (3.2)	7.3 (2.8)	7.3 (3.1)	7.8 (3.6)	7.6 (3.3)	7.8 (3.1)
Current smoker (%)	24	26	30	27	19	17
Medication use^a^ (%)	8.6	11.5	9.5	11.3	5.6	4.3
CRC lifestyle index and its components						
CRC lifestyle index^b^ (points)	2.0 (0.9)	1.1 (0.4)	1.8 (0.3)	2.1 (0.3)	2.5 (0.3)	3.0 (0.4)
Body fatness score (points)	0.50	0	0.50	0.75	0.75	1.00
BMI (kg/m^2^)	26 (6)	30 (6)	27 (6)	26 (5)	25 (5)	24 (3)
WC (cm)						
Women	82 (16)	96 (16)	85 (13)	80 (14)	77 (10)	75 (8)
Men	95 (15)	107 (15)	97 (11)	93 (14)	92 (11)	87 (9)
Height (cm)						
Women	163 (8)	167 (5)	163 (7)	162 (9)	162 (7)	159 (5)
Men	176 (9)	181 (6)	178 (7)	177 (10)	173 (9)	172 (5)
Inactive in leisure time (%)	19	41	24	14	9	0
Dietary score (points)	0.375	0.25	0.375	0.50	0.50	0.50
Whole grains (g/day)	49 (58)	35 (48)	44 (54)	52 (56)	59 (68)	67 (65)
Dairy products (g/day)	375 (371)	307 (307)	361 (374)	387 (342)	440 (360)	416 (405)
Red meat (g/week)	232 (500)	282 (605)	264 (447)	242 (413)	243 (500)	146 (372)
Processed meat (g/week)	175 (403)	196 (414)	189 (396)	140 (421)	174 (414)	169 (420)
Alcohol (100%, g/day)	6.3 (15)	9 (22)	6 (14)	6 (13)	6 (14)	5 (10)

*BMI* body mass index, *CRC* colorectal cancer, *IQR* interquartile range, *WC* waist circumference

^a^Use of metformin, psycholeptics, psychoanaleptics, proton pump inhibitors or constipation medication

^b^Higher points indicate a lower-risk lifestyle for CRC

The CRC lifestyle index was positively associated with alpha diversity (*β* 0.037, SE 0.013, *p* 0.009; Table 4). Of the index components, a higher BMI and WC were associated with a lower alpha diversity (BMI: *β* − 0.045, SE 0.013, *p* 0.002; WC: *β* − 0.048, SE 0.015, *p* 0.003), whereas being physically active versus inactive was associated with a higher diversity (*β* 0.091, SE 0.039, *p* 0.042). We observed no significant associations between the other components and alpha diversity.

**Table 4:** Associations of the CRC lifestyle index and its components with alpha diversity measured using the Shannon Index and beta diversity measured using the weighted UniFrac metric

	Alpha diversity Shannon Index			Beta diversity weighted UniFrac	
	*β*	SE	*p*^a^	*R*^2^	*p*^a^
CRC risk index^b,c^	0.037	0.013	0.009	0.002	0.010
Index components^d^					
Body fatness score	0.054	0.013	< 0.001	0.004	< 0.001
BMI (kg/m^2^)	− 0.045	0.013	0.002	0.005	< 0.001
WC (cm)	− 0.048	0.015	0.003	0.006	< 0.001
Height (cm)	0.023	0.019	0.33	0.002	0.039
Leisure-time physical activity (%)					
Inactive vs. active	0.091	0.039	0.042	0.003	0.10
Dietary score	0.008	0.013	0.69	0.002	0.007
Whole grains (g/day)	− 0.021	0.013	0.21	0.002	0.010
Dairy products (g/day)	0.008	0.013	0.75	0.001	0.052
Red meat (g/week)	− 0.015	0.013	0.37	0.0007	0.62
Processed meat (g/week)	− 0.003	0.013	0.86	0.002	0.003
Alcohol (100%) (g/day)	− 0.018	0.014	0.31	0.005	< 0.001

*BMI* body mass index, *CRC* colorectal cancer, *SE* standard error, *WC* waist circumference

^a^*p* values were corrected for multiple testing using the Benjamini–Hochberg false discovery rate (FDR)[53]

^b^Higher points indicate a lower-risk lifestyle for CRC

^c^Adjusted for sex, age, energy intake (MJ/day), smoking habits (smoker/nonsmoker), and use of potentially microbiome-altering medication (yes/no; metformin, psycholeptics, psychoanaleptics, proton pump inhibitors, and constipation medication)

^d^Adjusted for sex, age, energy intake (MJ/day), smoking habits (smoker/nonsmoker), use of potentially microbiome-altering medication (yes/no), and BMI (kg/m^2^; with predictors other than BMI and WC)

The CRC lifestyle index explained a small but statistically significant proportion of the variation in beta diversity (*R*^2^ 0.002, *p* 0.010; Table 4). Of the index components, all except for physical activity and the consumption of red meat and dairy products, were significantly associated with beta diversity. The explained proportions ranged from 0.1 to 0.6%, the largest proportions being for BMI, WC, and alcohol intake (0.5–0.6%). In dbRDA, the index together with the confounding variables (constrained variance) explained 1.4% (*p* 0.001) of the between-sample variation in beta diversity. The first two axes accounted for 76.9% of the constrained variance, and approximately 1% of the total variance (Online Resource 4). In qualitative interpretation, we identified a division along the second axis (RDA2) where male sex, energy intake, and smoking pointed in the opposite direction of the CRC lifestyle index and medication use. In PCoA, we observed no statistically significant differences in ordination patterns between participants in the extreme index quintiles (Q5 vs. Q1; *p* 0.17; Online Resource 5).

In the differential abundance analysis, we detected statistically significant associations between the CRC lifestyle index and 103 bacterial species (Online Resource 6). Of these, the majority were negative, indicating a higher relative abundance of species with lower index points. At the family level, negative associations were observed primarily with species within Lachnospiraceae, as well as with species from, for example, Coriobacteriaceae, Megasphaeraceae, and Erysipelotrichaceae. At the genus level, the negatively associated species represented, for example, *Dorea* (Lachnospiraceae), *Mediterraneibacter* (Lachnospiraceae), *Collinsella* (Coriobacteriaceae), and *Megasphaera* (Megasphaeraceae)*.* We observed positive associations between the index and genera *Bifidobacterium* (Bifidobacteriaceae; three species) and *Haemophilus* (Pasteurellaceae; three species), as well as with several species with incomplete taxonomy assignments across hierarchical levels.

**Fig. 2:**
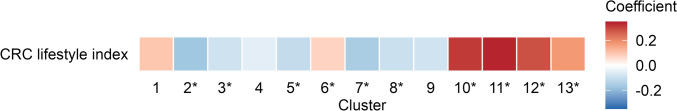
Associations between the CRC lifestyle index and 13 clusters composed of the species significantly associated with the index (*β* coefficient). An asterisk indicates a statistically significant association between the index and a cluster (*p* < 0.05). The clusters (C) included, for example, C1: 3 species from *Bifidobacterium*; C2: 11 species from, e.g., *Selenomonas* and *Megasphaera*; C3: 18 species from, e.g., *Dorea*, *Mediterraneibacter*, *Eubacterium*, and *Lachnoclostridium*; C4: 6 species from, e.g., *Megasphaera* and *Peptostreptococcus*; C5: 6 species from, e.g., *Mediterraneibacter*; C6: 8 species from, e.g., *Eubacterium*; C7: 6 species from, e.g., *Collinsella*; C8: 9 species from, e.g., *Porphyromonas*; C9: 10 species from, e.g., *Hungatella*, *Actinomyces*, *Lachnoclostridium*, and *Eubacterium*; C10: 3 species from *Haemophilus*; C11: 6 species, e.g., *Phil1 sp002069725*; C12: 5 species from *Cryptobacteroides*; C13: 12 species, e.g., *CAG-533 sp000434495*. See Online Resource 6 for further details on the clusters and species they comprise

In the cluster analysis, the bacterial species significantly associated with the CRC lifestyle index were divided into 13 clusters. Of these, five were positively and five negatively associated with the index (*p* < 0.05). Three clusters had a nonsignificant association, including a cluster formed of the three *Bifidobacterium* species (*p* > 0.05; Fig. 2, Online Resource 6). The positively associated clusters were highly diverse in terms of the included species, many of which were incompletely annotated. The negatively associated clusters were also diverse, although species in the family Lachnospiraceae were particularly prevalent. For example, cluster 3 included 18 species, of which majority were from Lachnospiraceae, such as *Mediterraneibacter* (three species), *Dorea* (two species), and *Lachnoclostridium* (one species).

## Discussion

This is the first study to explore associations between higher- and lower-risk lifestyles for CRC and the gut microbiome diversity and composition. CRC-related lifestyles were modelled using a CRC lifestyle index that demonstrated good applicability in Finnish adults. The index was associated with compositional differences in the gut microbiome between participants. Higher-risk lifestyles (lower index points) were associated with lower individual microbial diversity, higher relative abundance of bacterial species particularly in the family Lachnospiraceae, and lower relative abundance of species in the genus *Bifidobacterium*.

The CRC lifestyle index was adapted from the standardized 2018 WCRF/AICR Score [19] including nine major risk factors for CRC based on the 2018 WCRF/AICR Third Expert Report [20]. Previous prospective studies in the UK (*n* = 94,000; 863 CRC cases) [59], Spain (*n* = 7,216; 96 CRC cases) [60], and the US (*n* = 45,442 men; 1,151 CRC cases) [61] have reported a 21–48% lower CRC risk in participants with the highest versus lowest points in the standardized score or a similar index. Thus, our result of a 31% lower risk in Q5 versus Q1 fell within the range of these results, likely due to shared components across indices (BMI, red and processed meat consumption, alcohol intake). In general, the findings in prior studies corroborate our result, demonstrating that the CRC lifestyle index was an appropriate method to separate participants with higher- and lower-risk lifestyles for CRC in Finnish adults.

Despite growing interest in the gut microbiome’s role in CRC pathogenesis, research on the associations between CRC-related lifestyles and microbiome diversity is limited, and evidence on individual CRC risk factors in relation to alpha or beta diversity remains inconclusive. In a systematic review comparing participants with and without obesity, results on alpha diversity were varying with most studies reporting either nonsignificant association or lower diversity in participants with obesity (*n* = 20–1,674) [62]. BMI was, however, more consistently associated with beta diversity. Evidence on height is limited, although a positive association was observed with alpha diversity in an observational study of 3,409 US individuals participating in a commercial lifestyle intervention program [63]. Regarding physical activity, studies in a recent systematic review (*n* = 49–868) reported mainly nonsignificant differences in alpha diversity between highly active and inactive participants [64]. The results on beta diversity were inconsistent. In terms of dietary factors, observational studies (*n* = 222–1,135) have mostly reported nonsignificant associations between consumption of whole grains, dairy, red meat, processed meat, or alcohol and alpha diversity, whereas some significant associations have been observed with beta diversity [65–69].

We observed statistically significant associations between several index components and alpha and beta diversity. Although our results partly aligned with the previous findings, direct comparison is limited by differences in methods and study settings; for example, diverging dietary assessment methods and sample sizes may explain discrepancies. Furthermore, most of the above-mentioned studies relied on 16S ribosomal RNA (rRNA) instead of metagenomic sequencing [62–69]. Overall, the evidence of associations between individual CRC risk factors or whole lifestyles and microbial diversity is limited and inconsistent, highlighting the need for further research.

In the differential abundance analysis, we observed a higher relative abundance of species predominantly from the family Lachnospiraceae among those with lower index points (higher-risk lifestyles). Within Lachnospiraceae, these associations were apparent in genera such as *Dorea* (two species), *Lachnoclostridium* (two species) and *Mediterraneibacter* (four species), which have all been associated with CRC in previous studies [69–71]. Of these, the two *Dorea*, one *Lachnoclostridium*, and three *Mediterraneibacter* species were also assigned to the same cluster (cluster 3), indicating similar abundance patterns and co-occurrence among them. Additionally, *Dorea* has previously been linked to obesity [62], which suggests that BMI (and WC) contributed to its negative association with the CRC index. At the species level, we identified associations with, for example, *Dorea longicatena* and *Mediterraneibacter torques*, which have been found enriched in CRC patients compared with healthy controls in previous research [71, 72]. Within Lachnospiraceae, we also observed a negative association between the index and *Hungatella hathewayi*. *Hungatella hathewayi* has been linked with the key enzyme in the CRC-related pathway that produces trimethylamine (TMA) from choline, which is abundant in red meat and dairy [73].

Besides Lachnospiraceae, several species within families such as Erysipelotrichaceae, Coriobacteriaceae, and Megasphaeraceae, were negatively associated with the index. In a cross-sectional study of 102 Chinese adults (46 CRC patients and 56 healthy controls), both Erysipelotrichaceae and Coriobacteriaceae were enriched in CRC patients compared with healthy controls [74]. Within Coriobacteriaceae, particularly genus *Collinsella* has been linked to CRC [70, 74]. We identified four *Collinsella* species (*C. intestinalis, C. stercoris*, *C. ihuae*, and *C. phocaeensis*) that were negatively associated with the index. These four, along with two other Coriobacteriaceae species (*Enorma sp000333815* and *Limicola sp002160065*), were also clustered together (cluster 7). Furthermore, Coriobacteriaceae, Erysipelotrichaceae, and the genus *Megasphaera* within Megasphaeraceae have previously been linked to obesity [62, 74].

We observed positive associations between the CRC lifestyle index and three *Bifidobacterium* species (*B. longum*, *B. saguini*, and *B. breve*). In previous studies, a reduced abundance of *Bifidobacterium* has been associated with CRC particularly in the early disease stages [72, 74]. *Bifidobacterium* has also been negatively associated with obesity [75]. In contrast, high intake of dietary fiber appears to increase the abundance of *Bifidobacterium,* which is likely driven by *Bifidobacterium* being one of the genera that ferments fiber into short-chain fatty acids, SCFAs [76]. SCFAs, in turn, have several properties related, for example, to the regulation of inflammation and epigenetic mechanisms, which may benefit the prevention of colorectal carcinogenesis [3].

Overall, our results suggest that several bacterial genera and species that have been previously linked to CRC are also associated with high-risk lifestyles for CRC. Nevertheless, we did not observe significant associations with the species most consistently linked to CRC, such as *Fusobacterium nucleatum* and enterotoxigenic *Bacteroides fragilis* [4, 5]. This may be due to variation in microbial composition over the progression of carcinogenesis. For example, in a study of 616 Japanese adults, *F. nucleatum* was linked particularly with advanced CRC stages [72]. In a study of 500 German adults, *F. nucleatum* was associated with CRC but neither with pre-cancerous lesions in the gut nor with diet or other lifestyle factors [77]. As our study sample included apparently healthy adults with no CRC diagnosis at baseline, and only one incident case over the first 8 years of follow-up, our results provide information on microbial traits that potentially characterize pre-cancerous stages of CRC development in high-risk individuals. While our results support previous research linking several microbial taxa to CRC, further research is needed to better understand the shifts in the gut microbiome throughout CRC progression, from pre-cancerous to more advanced stages. Furthermore, the functional characterization of the species associated with higher-risk lifestyles for CRC remains an important direction for future research.

Our results should be interpreted considering the study limitations. First, lifestyle data were self-reported, introducing potential bias, especially in dietary intake due to misreporting. This typically attenuates associations between dietary intake and health outcomes. In addition, different dietary assessment methods were used in testing the index (FFQ) and analyzing associations with the microbiome (48-h recall), which may have caused some discrepancy in the index performance. However, because we focused on commonly consumed foods in Finland, the recall data likely reasonably reflected participants’ habitual dietary intake [23, 26, 78]. Diet was also only one of the index components. Another consideration related to the index is that, although including whole grains over dietary fiber in the index was well-justified for our study population, fiber intake may be a more relevant factor in other populations where whole grain intake is lower. Regarding the study population, in the pooled cohorts, most participants were men, which may limit the generalizability of those findings regarding the index performance overall and with respect to the microbiome analyses. However, we observed no between-cohort heterogeneity, and the results in the individual cohorts were parallel between sexes. Furthermore, in FINRISK/FINDIET 2002 used in the microbiome analyses, the study population was well balanced between men (46% of the study population) and women.

In the microbiome analyses, we included individuals who donated a fecal sample and participated in the FINDIET substudy. These participants were likely more health-conscious compared to non-participants. Moreover, prior findings from FINRISK 2002 suggested that the sample donors were on average older, more likely women, less likely smokers, and had healthier diets than non-donors [79]. Thus, our study population may not fully represent the general Finnish adult population. However, this bias is likely reduced by the reasonably high participation rate in FINRISK/FINDIET 2002 (64%). Regarding the adjustment for constipation medication and proton-pump inhibitors, we could only account for prescription-based use, for which non-prescription use may have been overlooked. The limitations of shallow sequencing, compared with deep sequencing, in capturing genetic features and low-abundance taxa should also be acknowledged. However, we anticipate that these limitations are unlikely to substantially affect the alpha and beta diversity analyses and findings, or the differential abundance of species-level taxa presented here [42]. Overall, our results should be primarily interpreted as exploratory and descriptive rather than predictive. For example, although statistically significant, the index and its components explained only very small proportions of the between-individual variation in microbial composition. Such modest effect sizes are typical in microbiome research and reflect the many environmental and host factors that shape microbial communities, but their predictive or clinical relevance is likely limited. Finally, the cross-sectional study design limits our ability to draw conclusions about causality.

This study also has several strengths. We tested the CRC lifestyle index in a large, pooled data of five Finnish cohorts with long median follow-up and extensive information on participants’ lifestyle and health, including a validated FFQ. Information on CRC cases was obtained from the Finnish Cancer Registry, which has maintained comprehensive nationwide records of all cancer diagnoses in Finland since 1953 [40]. In the microbiome analyses, we used a large population-based study sample with comprehensive lifestyle and health data, which allowed adjustment for key confounders, including several types of medication. Furthermore, we excluded participants with a recent purchase of systemic antibacterial medication. Finally, the microbiome data were based on whole-metagenome sequencing, which provides more accurate taxonomic identification and classification than 16S rRNA gene amplicon-based sequencing [42].

To conclude, our results suggested that the diversity and composition of the gut microbiome differ between participants with higher- and lower-risk lifestyles for CRC. Higher-risk lifestyles were associated with a higher relative abundance of several microbial species previously linked to CRC. Enrichment in these species may reflect microbial alterations contributing to the initiation or progression of CRC carcinogenesis. Overall, our findings add to the current evidence of the interplay between lifestyle, the gut microbiome, and CRC risk, providing insight into microbial traits that may characterize high-risk individuals.

## Supplementary Information

Below is the link to the electronic supplementary material.

Supplementary file1 (PDF 562 kb)

## Data Availability

The datasets analyzed during the current study will be made available upon request pending application and approval through the Findata Permit Procedure at https://www.findata.fi/en/ or through a written application to the Finnish Institute for Health and Welfare Biobank (https://thl.fi/en/web/thl-biobank).
