# Supplementary material for: Interplay between colorectal cancer-related lifestyles and the gut microbiome: an exploratory analysis of metagenomic data
Source: Cancer Causes Control. 2026 Mar 11;37(4):58. doi: 10.1007/s10552-026-02144-1 (PMC12979301; doi:10.1007/s10552-026-02144-1)
Supplement: Supplementary file 1 — Supplementary file1 (PDF 562 kb) [file 10552_2026_2144_MOESM1_ESM.pdf]

Supplementary Information

Cancer Causes & Control

Interplay between colorectal cancer-related lifestyles and the gut microbiome: an exploratory analysis of metagenomic data

Rilla Tammi, Mirkka Maukonen, Niina E Kaartinen, Kari K Koponen, Teemu Niiranen, Guillaume Méric, Demetrius Albanes, Johan G Eriksson, Pekka Jousilahti, Seppo Koskinen, Anne-Maria Pajari, Rob Knight, Aki S Havulinna, Veikko Salomaa, Satu Männistö

Corresponding author

Rilla Tammi

Finnish Institute for Health and Welfare (THL), P.O. Box 30, 00271, Helsinki, Finland

rilla.tammi@thl.fi

ORCID: 0000-0002-5507-2314

This supplementary information includes six online resources:

Online Resource 1: a table of the characteristics of the pooled cohorts used in testing the CRC lifestyle index
Online Resource 2: a flow chart of the exclusions made in each pooled cohort
Online Resource 3: a table of background factors and the CRC lifestyle index components in the pooled cohorts
Online Resource 4: a dbRDA ordination plot
Online Resource 5: a PCoA ordination plot
Online Resource 6: a table of associations of the CRC lifestyle index with species-level taxa and their corresponding clusters

Online Resource 1 Characteristics of the pooled cohorts used to test the CRC lifestyle index

Cohort	Baseline years	Baseline age, years	Final number of participants[a]	Number of men (%)	End year of follow-up	Median follow-up time, years	Number of incident CRC cases
ATBC (13)	1984–1988	50–70	26,915	26,915 (100%)	2016	17.9	948
Health 2000 (14)	2000–2001	30–99	5,565	2,522 (45%)	2015	15.2	67
HBCS (15)	2001–2004	56–69	1,853	870 (47%)	2014	12.0	36
DILGOM 2007 (16)	2007	25–74	4,651	2,167 (47%)	2019	12.6	46
FINRISK 2012 (17)	2012	25–74	4,504	2,066 (46%)	2019	7.8	21
Total			43,488	34,540 (79%)		14.0	1,118

CRC, colorectal cancer; ATBC, the Alpha-Tocopherol, Beta-Carotene Cancer Prevention Study; Health 2000, the Health 2000 Health Examination Survey; HBCS, the Helsinki Birth Cohort Study; DILGOM 2007, the DIetary, Lifestyle and Genetic Determinants of Obesity and Metabolic Syndrome 2007 Study; FINRISK 2012, the National FINRISK 2012 Study

[a] Exclusion criteria: missing or inadequately filled FFQ (incomplete questionnaire with several empty rows, exclusions made case by case), implausible energy intake (ATBC: <1,000 or >5,000 kcal/d; Health 2000: <600 or >7,000 kcal/d; HBCS, DILGOM 2007 and FINRISK 2012: 0.5% sex-specific extremes in energy intake distribution); history of cancer at baseline (excluding nonmelanoma skin cancer); missing information on CRC lifestyle index components (body mass index, waist circumference [not measured in ATBC], height, leisure-time physical activity, consumption of whole grains, dairy products, red meat and processed meat, and alcohol intake).

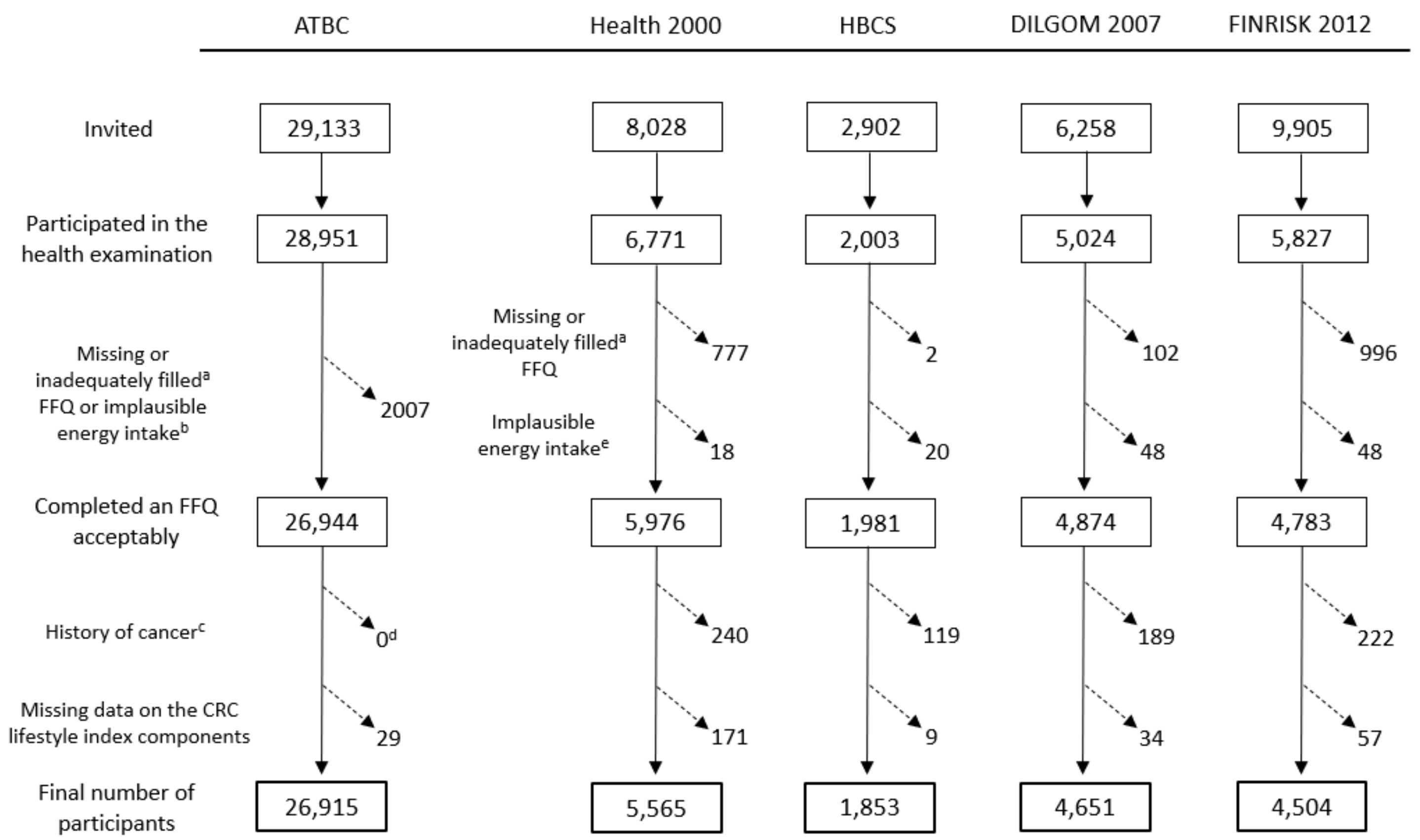

Online Resource 2 Exclusions and final study samples of the pooled cohorts used to test the CRC lifestyle index

ATBC, the Alpha-Tocopherol, Beta-Carotene Cancer Prevention Study; Health 2000, the Health 2000 Health Examination Survey; HBCS, the Helsinki Birth Cohort Study; DILGOM 2007, the DIetary, Lifestyle and Genetic Determinants of Obesity and Metabolic Syndrome 2007 Study; FINRISK 2012, the National FINRISK 2012 Study; FFQ, food frequency questionnaire

[a] Incomplete questionnaire with several empty food item rows (exclusions made case by case)

[b] Energy intake <1,000 or >5,000 kcal/d

[c] History of cancer other than nonmelanoma skin cancer

[d] Individuals with history of cancer (other than nonmelanoma skin cancer) were excluded in the recruitment process.

[e] Health 2000: energy intake <600 or >7,000 kcal/d; HBCS, DILGOM 2007 and FINRISK 2012: 0.5% sex-specific extremes in energy intake distribution

Online Resource 3 Background factors and the CRC lifestyle index components in total and by the index quintiles (medians [IQR] or proportions [%]) in the pooled cohorts

		CRC lifestyle index quintiles			
	Total	Q1	Q3	Q5	P_{trend}[a,b]
Background factors, median (IQR) or %					
Number of men (%)					
ATBC[c]	26,915 (100%)	4,970 (100%)	5,164 (100%)	6,222 (100%)	
Health 2000	2,522 (45%)	489 (45%)	534 (45%)	611 (50%)	
HBCS	870 (47%)	178 (48%)	170 (46%)	199 (51%)	
DILGOM 2007	2,167 (47%)	412 (46%)	406 (45%)	423 (48%)	
FINRISK 2012	2,066 (46%)	438 (46%)	425 (46%)	366 (45%)	
Age, years					
ATBC	57.1 (8.0)	55.7 (7.2)	57.1 (7.7)	58.4 (8.1)	<0.0001
Health 2000	50.0 (21.0)	48.0 (16.0)	50.0 (21.0)	51.0 (23.0)	<0.0001
HBCS	60.0 (4.0)	60.0 (4.0)	60.0 (5.0)	61.0 (5.0)	<0.0001
DILGOM 2007	52.6 (21.7)	51.1 (19.7)	54.6 (22.5)	53.0 (23.7)	0.58
FINRISK 2012	53.0 (24.0)	53.0 (19.0)	54.0 (22.2)	53.0 (28.0)	0.002
Energy intake, MJ/d					
ATBC	10.8 (4.0)	10.8 (4.2)	10.5 (4.0)	10.9 (3.8)	0.99
Health 2000	9.1 (3.9)	8.7 (3.9)	8.8 (3.7)	9.4 (4.0)	<0.0001
HBCS	8.7 (4.1)	8.3 (4.1)	8.7 (4.0)	9.0 (4.0)	0.009
DILGOM 2007	9.9 (4.5)	9.4 (4.7)	9.9 (4.5)	10.3 (4.6)	0.0001
FINRISK 2012	8.9 (4.1)	8.5 (4.4)	9.0 (4.2)	9.2 (3.6)	0.014
Low educational attainment, %					
ATBC	78	71	77	85	<0.0001
Health 2000	32	35	31	30	0.20
HBCS	33	38	32	32	0.20
DILGOM 2007	30	30	31	29	0.95
FINRISK 2012	33	37	34	29	0.0006
Current smokers, %					
ATBC[c]	100	100	100	100	
Health 2000	26	32	27	22	0.0002
HBCS	24	26	21	24	0.70
DILGOM 2007	18	22	18	14	<0.0001
FINRISK 2012	17	24	14	14	<0.0001
Prevalent diabetes, %					
ATBC	6	10	5	4	<0.0001
Health 2000	4	5	4	2	0.0003
HBCS	2	3	1	1	0.033
DILGOM 2007	9	12	9	6	<0.0001
FINRISK 2012	12	18	12	6	<0.0001
CRC lifestyle index and its components, median (IQR) or %					
CRC lifestyle index, points					
ATBC	1.875	1.000	1.875	2.625	
Health 2000	1.875	1.000	1.875	2.750	
HBCS	1.750	0.875	1.750	2.750	
DILGOM 2007	2.125	1.125	2.125	3.000	
FINRISK 2012	2.000	1.125	2.000	3.000	
Body fatness score, points					
ATBC	0.50	0.50	0.50	1.00	
Health 2000	0.50	0.25	0.50	1.00	
HBCS	0.50	0	0.50	1.00	
DILGOM 2007	0.50	0.25	0.50	1.00	

FINRISK 2012	0.50	0	0.50	1.00
Body mass index, kg/m^2				
ATBC	26 (5)	29 (5)	26 (4)	24 (3)
Health 2000	26 (6)	30 (5)	26 (5)	24 (4)
HBCS	27 (5)	30 (5)	27 (5)	24 (3)
DILGOM 2007	26 (6)	30 (6)	26 (6)	24 (3)
FINRISK 2012	26 (6)	30 (6)	27 (6)	24 (3)
Waist circumference[d], cm				
Women				
Health 2000	87 (18)	98 (15)	86 (16)	77 (10)
HBCS	90 (17)	101 (15)	90 (13)	79 (11)
DILGOM 2007	84 (17)	97 (16)	85 (14)	76 (7)
FINRISK 2012	86 (19)	98 (18)	87 (16)	76 (9)
Men				
Health 2000	97 (15)	108 (12)	97 (10)	89 (9)
HBCS	100 (14)	108 (12)	100 (11)	92 (9)
DILGOM 2007	96 (15)	107 (13)	97 (11)	87 (8)
FINRISK 2012	97 (17)	109 (14)	98 (13)	88 (10)
Height, cm				
Women[e]				
Health 2000	163 (9)	167 (6)	163 (9)	158 (7)
HBCS	163 (8)	167 (6)	163 (7)	159 (5)
DILGOM 2007	163 (9)	167 (5)	162 (9)	159 (6)
FINRISK 2012	163 (9)	167 (7)	163 (8)	159 (5)
Men				
ATBC	174 (9)	178 (5)	173 (7)	169 (5)
Health 2000	176 (10)	181 (6)	176 (9)	171 (7)
HBCS	177 (8)	181 (4)	177 (8)	173 (5)
DILGOM 2007	176 (9)	181 (7)	175 (9)	171 (5)
FINRISK 2012	177 (9)	181 (6)	177 (9)	172 (5)
Inactive in leisure time, %				
ATBC	42	77	39	14
Health 2000	27	57	22	4
HBCS	31	66	26	3
DILGOM 2007	19	55	10	0
FINRISK 2012	20	53	14	0
Dietary score, points				
ATBC	0.50	0.50	0.50	0.625
Health 2000	0.50	0.375	0.50	0.50
HBCS	0.375	0.375	0.375	0.50
DILGOM 2007	0.50	0.375	0.50	0.50
FINRISK 2012	0.50	0.375	0.50	0.50
Whole grains, g/d				
ATBC	100 (85)	83 (90)	83 (74)	116 (76)
Health 2000	58 (58)	54 (46)	57 (52)	69 (45)
HBCS	54 (51)	42 (40)	52 (49)	65 (35)
DILGOM 2007	76 (63)	61 (59)	79 (52)	84 (54)
FINRISK 2012	69 (65)	57 (57)	70 (63)	78 (66)
Dairy products, g/d				
ATBC	699 (502)	651 (614)	653 (508)	737 (448)
Health 2000	543 (466)	456 (497)	530 (445)	595 (435)
HBCS	436 (434)	340 (394)	396 (443)	552 (441)
DILGOM 2007	583 (507)	457 (487)	568 (521)	629 (488)
FINRISK 2012	579 (515)	484 (511)	602 (512)	664 (485)
Red meat, g/week				
ATBC	456 (274)	474 (276)	445 (270)	448 (273)
Health 2000	514 (346)	520 (326)	516 (338)	515 (373)
HBCS	419 (346)	449 (378)	420 (348)	391 (348)
DILGOM 2007	513 (409)	543 (405)	514 (406)	483 (434)
FINRISK 2012	462 (354)	497 (367)	474 (342)	433 (355)

Processed meat, g/week				
ATBC	421 (403)	458 (428)	411 (404)	406 (394)
Health 2000	253 (338)	272 (341)	235 (318)	229 (318)
HBCS	211 (279)	237 (317)	228 (288)	188 (262)
DILGOM 2007	281 (352)	315 (431)	275 (325)	267 (357)
FINRISK 2012	268 (341)	298 (355)	274 (335)	227 (288)
Alcohol (100%), g/d				
ATBC	11.0 (23.0)	17.5 (29.1)	17.5 (27)	7.0 (16.2)
Health 2000	2.5 (6.7)	2.7 (6.9)	2.8 (6.6)	2.3 (5.8)
HBCS	4.9 (10.6)	5.1 (13.2)	5.7 (10.7)	3.4 (10.0)
DILGOM 2007	3.8 (9.1)	4.5 (9.9)	3.5 (8.9)	3.6 (7.8)
FINRISK 2012	3.8 (9.4)	3.6 (10.0)	3.9 (9.6)	3.8 (8.1)

CRC, colorectal cancer; IQR, interquartile ranges; ATBC, the Alpha-Tocopherol, Beta-Carotene Cancer Prevention Study; Health 2000, the Health 2000 Health Examination Survey; HBCS, the Helsinki Birth Cohort Study; DILGOM 2007, the DIetary, Lifestyle and Genetic Determinants of Obesity and Metabolic Syndrome 2007 Study; FINRISK 2012, the National FINRISK 2012 Study; HRT, hormone replacement therapy

[a] *P* for trend was tested using score medians for the index quintiles as continuous independent variables.

[b] Adjusted for sex, age, energy intake, educational attainment, and smoking. A variable was excluded from adjustments if it was used as an outcome variable.

[c] All participants in ATBC were male current smokers as per the study design.

[d] Waist circumference was not measured in ATBC.

[e] ATBC only included men.

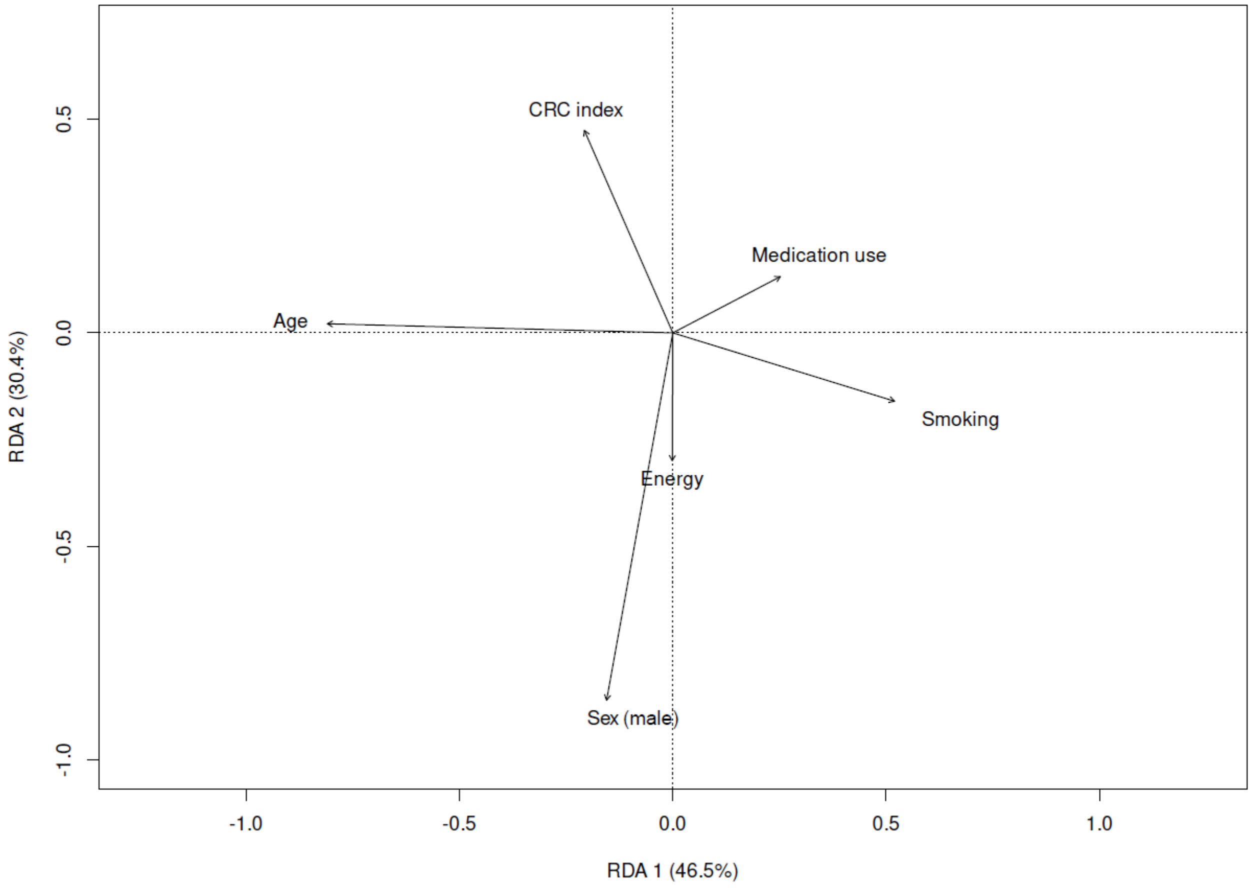

Online Resource 4 Directions of associations between the CRC lifestyle index, confounding variables, and beta diversity (weighted UniFrac metric) based on the distance-based redundancy analysis (dbRDA). DbRDA visualizes the variation in distances between samples regarding beta diversity while constraining their ordination with the chosen variables. The vectors display the direction, the strength of association (vector length), and relationship between variables (angle between vectors, how similar is the effect) in the associations of the CRC lifestyle index and confounding variables with beta diversity on the first two axes of the ordination. The first two axes (RDA 1 and RDA 2) together explained 76.9% of the variance in beta diversity.

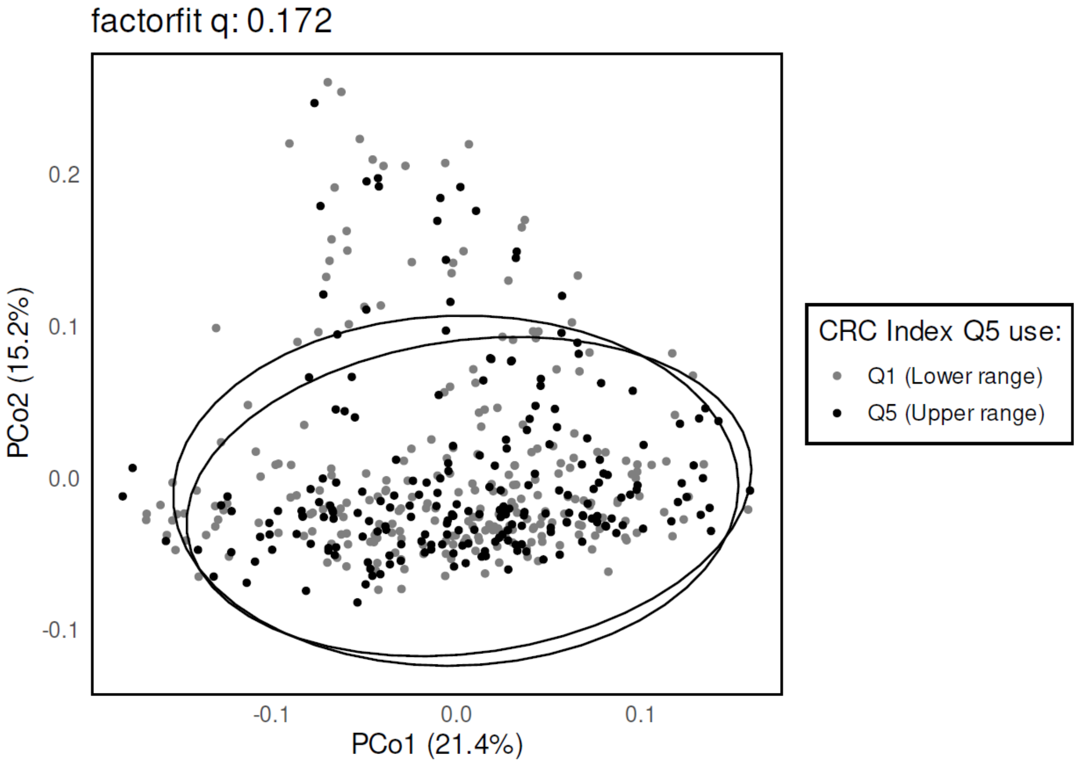

Online Resource 5 Ordination of the first and second beta-diversity (weighted UniFrac) principal components between the participants with the highest (Q5) and lowest (Q1) CRC lifestyle index points. The False-Discovery Rate corrected p-value from the factorfit-test (factorfit q) indicate whether there are statistically significant differences in ordination between participants in the extreme quintiles of the index. The axis labels (PCo1 and PCo2) indicate the proportion of variation explained by each principal component.

Online Resource 6 Associations of the CRC lifestyle index with species-level taxa and their corresponding clusters

	CRC lifestyle index		
Phylum/Class/Order/Family/**Genus Species**	*Effect size*[a]	*SE*	*P*[b]
**Cluster 1**	0.098	0.056	0.081
*Actinomycetota/Actinomycetes/Bifidobacteriales/Bifidobacteriaceae/**Bifidobacterium longum***	0.141	0.00007	<0.0001
*Actinomycetota/Actinomycetes/Bifidobacteriales/Bifidobacteriaceae/**Bifidobacterium saguini***	0.070	0.00005	<0.0001
*Actinomycetota/Actinomycetes/Bifidobacteriales/Bifidobacteriaceae/**Bifidobacterium breve***	0.084	0.00006	<0.0001
**Cluster 2**	**-0.173**	**0.056**	**0.002**
*Bacillota/Bacilli/Lactobacillales/Lactobacillaceae/**Lactobacillus gasseri_329736***	-0.084	0.00004	<0.0001
*Pseudomonadota/Gammaproteobacteria/Burkholderiales/Burkholderiaceae/**Lautropia mirabilis***	0.123	0.00003	<0.0001
*Bacillota/Negativicutes/Selenomonadales /Selenomonadaceae/**Selenomonas sputigena***	-0.153	0.00002	<0.0001
*Bacillota/Negativicutes/Acidaminococcales/Acidaminococcaceae/**Acidaminococcus intestini***	-0.210	0.00007	<0.0001
*Pseudomonadota/Gammaproteobacteria/Enterobacterales/Pasteurellaceae/**Necropsobacter massiliensis***	0.255	0.00003	<0.0001
*Bacillota/Negativicutes/Veillonellales/Megasphaeraceae/**Megasphaera_A_38685 cerevisiae***	-0.307	0.00004	<0.0001
*Actinomycetota/Coriobacteriia/Coriobacteriales/Atopobiaceae/**UBA7741 sp900314495***	-0.017	0.00003	<0.0001
*Bacillota/Negativicutes/Veillonellales/Megasphaeraceae/**Caecibacter sp003467125***	-0.167	0.00005	<0.0001
*Bacteroidota/Bacteroidia/Bacteroidales/Dysgonomonadaceae/**Petrimonas sp012728465***	-0.116	0.00003	<0.0001
*Bacillota/Negativicutes/Veillonellales/Dialisteraceae/**Allisonella histaminiformans***	-0.256	0.00006	<0.0001
*Bacillota/Clostridia/Oscillospirales/Acutalibacteraceae/**UBA6857 sp902792985***	0.082	0.00004	<0.0001
**Cluster 3**	**-0.091**	**0.037**	**0.015**
*Bacillota/Clostridia/Lachnospirales/Lachnospiraceae/**Agathobacter rectalis***	-0.166	0.00006	<0.0001
*Bacillota/Clostridia/Lachnospirales/Lachnospiraceae/**Dorea_A longicatena***	-0.100	0.00004	<0.0001
*Bacillota/Clostridia/Lachnospirales/Lachnospiraceae/**Bariatricus comes***	-0.106	0.00004	<0.0001
*Bacillota/Bacilli/Erysipelotrichales/Erysipelotrichaceae/**Holdemania sp900120005***	-0.113	0.00004	<0.0001
*Bacillota/Clostridia/Lachnospirales/Lachnospiraceae/**Dorea_A formicigenerans***	-0.080	0.00003	<0.0001
*Bacillota/Clostridia/Lachnospirales/Lachnospiraceae/**14-2 sp000403255***	-0.080	0.00004	<0.0001
*Bacillota/Clostridia/Lachnospirales/Lachnospiraceae/**Eubacterium_I ramulus***	-0.092	0.00004	<0.0001
*Bacillota/Clostridia/Lachnospirales/Lachnospiraceae/**Mediterraneibacter_A_155507 faecis***	-0.085	0.00004	<0.0001

*Bacillota/Clostridia/Lachnospirales/Lachnospiraceae/**Mediterraneibacter_A_155507 massiliensis***	-0.080	0.00003	<0.0001
*Bacillota/Clostridia/Lachnospirales/Lachnospiraceae/**Mediterraneibacter_A_155590 butyricigenes***	-0.072	0.00003	<0.0001
*Bacillota/Clostridia/Lachnospirales/Lachnospiraceae/**Enterocloster sp000431375***	-0.243	0.00004	<0.0001
*Unclassified/**CAG-317 sp000433215***	-0.059	0.00003	<0.0001
*Bacillota/Clostridia/Eubacteriales/unclassified/**Ventrimonas sp003481825***	-0.054	0.00003	<0.0001
*Pseudomonadota/Gammaproteobacteria/Enterobacterales/Succinivibrionaceae/**Succinivibrio hippei_B***	-0.080	0.00004	<0.0001
*Bacillota/Clostridia/Lachnospirales/Lachnospiraceae/**Lachnoclostridium_B sp900066555***	-0.079	0.00003	<0.0001
*Bacillota/Clostridia/Lachnospirales/Lachnospiraceae/**Hespellia stercorisuis***	-0.074	0.00003	<0.0001
*Unclassified/**Copromonas sp000435795***	-0.129	0.00004	<0.0001
*Unclassified/**BX12 sp902363595***	-0.069	0.00003	<0.0001
**Cluster 4**	-0.052	0.044	0.235
*Bacillota/Clostridia/Lachnospirales/Lachnospiraceae/**Lacrimispora saccharolytica***	-0.100	0.00004	<0.0001
*Bacillota/Clostridia/Lachnospirales/Lachnospiraceae/**Lachnoanaerobaculum saburreum***	-0.093	0.00004	<0.0001
*Bacillota/Negativicutes/Veillonellales/Megasphaeraceae/**Megasphaera_A 38692 hutchinsoni***	-0.073	0.00003	<0.0001
*Bacillota/Clostridia/Peptostreptococcales/Peptostreptococcaceae/**Peptostreptococcus anaerobius***	-0.066	0.00003	<0.0001
*Bacillota/Clostridia/Peptostreptococcales/Anaerovoracaceae/**S5-A14a sp000758905***	-0.057	0.00003	<0.0001
*Bacillota/Clostridia/Lachnospirales/Lachnospiraceae/**F0428 sp003043955***	-0.064	0.00003	<0.0001
**Cluster 5**	**-0.111**	**0.039**	**0.004**
*Bacillota/Clostridia/Lachnospirales/Lachnospiraceae/**Mediterraneibacter_A 155507 torques***	-0.263	0.00005	<0.0001
*Bacillota/Clostridia/Oscillospirales/Oscillospiraceae/**Dysosmobacter welbionis***	-0.139	0.00004	<0.0001
*Bacillota/Clostridia/Oscillospirales/Oscillospiraceae/**Lawsonibacter asaccharolyticus***	-0.151	0.00004	<0.0001
*Bacillota/Clostridia/Oscillospirales/Oscillospiraceae/**Flavonifractor plautii***	-0.251	0.00006	<0.0001
*Unclassified/**Phocea massiliensis***	-0.130	0.00004	<0.0001
*Unclassified/**An92 sp900199495***	-0.118	0.00004	<0.0001
**Cluster 6**	**0.074**	**0.036**	**0.039**
*Bacillota/Clostridia/Lachnospirales/Lachnospiraceae/**Butyrivibrio_A_180067 crossotus***	0.115	0.00006	<0.0001
*Bacillota/Bacilli/Erysipelotrichales/Erysipelotrichaceae/**Holdemanella biformis***	-0.111	0.00005	<0.0001
*Bacteroidota/Bacteroidia/Bacteroidales/Rikenellaceae/**Alistipes_A_871400 senegalensis***	0.114	0.00005	<0.0001
*Bacillota/Clostridia/Oscillospirales/Oscillospiraceae/**Intestinimonas massiliensis***	0.086	0.00003	<0.0001
*Patescibacteria/Paceibacteria/Paceibacterales/UBA5633/**MWCK01 sp002070005***	-0.192	0.00009	<0.0001

*Bacillota/Clostridia/Lachnospirales/Lachnospiraceae/**Acetatifactor sp900066565***	0.112	0.00005	<0.0001
*Bacillota/Clostridia/Lachnospirales/Lachnospiraceae/**Eubacterium_F sp003491505***	0.207	0.00006	<0.0001
*Bacillota/Clostridia/Lachnospirales/CAG-274/**CAG-274 sp900545305***	-0.182	0.00009	<0.0001
**Cluster 7**	**-0.155**	**0.070**	**0.028**
*Actinomycetota/Coriobacteriia/Coriobacteriales/Coriobacteriaceae/**Collinsella intestinalis***	-0.142	0.00005	<0.0001
*Actinomycetota/Coriobacteriia/Coriobacteriales/Coriobacteriaceae/**Collinsella stercoris***	-0.124	0.00005	<0.0001
*Actinomycetota/Coriobacteriia/Coriobacteriales/Coriobacteriaceae/**Enorma sp000333815***	-0.092	0.00004	<0.0001
*Actinomycetota/Coriobacteriia/Coriobacteriales/Coriobacteriaceae/**Limicola sp002160065***	-0.015	0.00003	<0.0001
*Actinomycetota/Coriobacteriia/Coriobacteriales/Coriobacteriaceae/**Collinsella ihuae***	-0.076	0.00004	<0.0001
*Actinomycetota/Coriobacteriia/Coriobacteriales/Coriobacteriaceae/**Collinsella phocaeensis***	-0.067	0.00004	<0.0001
**Cluster 8**	**-0.095**	**0.045**	**0.034**
*Bacteroidota/Bacteroidia/Bacteroidales/Bacteroidaceae/**Prevotella salivae***	-0.089	0.00005	<0.0001
*Bacillota/Clostridia/Lachnospirales/Anaerotignaceae/**Coprocola sp000364165***	-0.032	0.00003	<0.0001
*Bacillota/Bacilli/Lactobacillales/Enterococcaceae/**Enterococcus_E cecorum***	-0.078	0.00004	<0.0001
*Bacteroidota/Bacteroidia/Bacteroidales/Prophyromonadaceae/**Porphyromonas_A_859423 pasteri***	-0.045	0.00005	<0.0001
*Bacteroidota/Bacteroidia/Bacteroidales/Prophyromonadaceae/**Porphyromonas_A_859426 somerae***	-0.173	0.00005	<0.0001
*Patescibacteria/Saccharimonadia/Saccharimonadales/Nanogingivalaceae/**Nanogingivalis sp010014525***	-0.058	0.00003	<0.0001
*Bacteroidota/Bacteroidia/Bacteroidales/Marinifilaceae/**Butyricimonas synergistica***	-0.093	0.00005	<0.0001
*Bacillota/Bacilli/Lactobacillales/Listeriaceae/**Listeria_A costaricensis***	-0.192	0.00004	<0.0001
*Bacillota/Clostridia/Peptostreptococcales/Anaerovoracaceae/**Bacilliculturomica massiliensis***	-0.073	0.00003	<0.0001
**Cluster 9**	-0.086	0.045	0.053
*Bacillota/Clostridia/Lachnospirales/Lachnospiraceae/**Hungatella_A_127239 hathewayi_A***	-0.107	0.00004	<0.0001
*Actinomycetota/Actinomycetes/Actinomycetales/Actinomycetaceae/**Actinomyces graevenitzii***	-0.058	0.00004	<0.0001
*Bacillota/Clostridia/Lachnospirales/Lachnospiraceae/**Lachnoclostridium_A_130679 sp003464085***	-0.093	0.00003	<0.0001
*Bacillota/Bacilli/Erysipelotrichales/Erysipelotrichaceae/**Absicoccus porci***	-0.102	0.00004	<0.0001
*Actinomycetota/Coriobacteriia/Coriobacteriales/Eggerthellaceae/**Slackia_A equolifaciens***	-0.071	0.00003	<0.0001
*Bacillota/Clostridia/Lachnispirales/Lachnospiraceae/**Cuneatibacter caecimuris***	-0.107	0.00004	<0.0001
*Bacillota/Bacilli/Lactobacillales/Streptococcaceae/**Streptococcus halichoeri***	-0.075	0.00003	<0.0001
*Bacillota/Clostridia/Lachnospirales/Lachnospiraceae/**Fimimorpha sp900045905***	-0.033	0.00003	<0.0001
*Bacillota/Clostridia/Peptostreptococcales/Anaerovoracaceae/**Eubacterium_T pyruvativorans***	-0.060	0.00004	<0.0001

*Bacillota/Bacilli/Lactobacillales/Streptococcaceae/***Streptococcus sp902729355**	-0.011	0.00003	<0.0001
**Cluster 10**	**0.314**	**0.096**	**0.001**
*Pseudomonadota/Gammaproteobacteria/Enterobacterales/Pasteurellaceae/***Haemophilus_A sputorum**	0.139	0.00005	<0.0001
*Pseudomonadota/Gammaproteobacteria/Enterobacterales/Pasteurellaceae/***Haemophilus_D_735815 parainfluenzae K 735050**	0.157	0.00007	<0.0001
*Pseudomonadota/Gammaproteobacteria/Enterobacterales/Pasteurellaceae/***Haemophilus_D_735815 parainfluenzae_K 735055**	0.233	0.00007	<0.0001
**Cluster 11**	**0.338**	**0.119**	**0.004**
*Bacillota/Clostridia/Christensenellales/CAG-138/***Phil1 sp002069725**	0.083	0.00003	<0.0001
*Unclassified/***QALA01 sp003343565**	0.008	0.00004	<0.0001
*Bacillota/Clostridia/Christensenellales/CAG-917/***CAG-475 sp900548005**	0.106	0.00005	<0.0001
*Bacillota/Bacilli_A/RF39/UBA660/***CAG-1000 sp902764145**	0.061	0.00003	<0.0001
*Bacillota/Clostridia/Christensenellales/CAG-138/***RUG472 sp902765005**	0.197	0.00005	<0.0001
*Bacillota/Bacilli/RF39/UBA660/***CAG-417 sp902791625**	0.086	0.00004	<0.0001
**Cluster 12**	**0.284**	**0.078**	**0.0003**
*Bacteroidota/Bacteroidia/Bacteroidales/UBA932/***Cryptobacteroides sp002438635**	0.216	0.00004	<0.0001
*Bacteroidota/Bacteroidia/Bacteroidales/UBA932/***Cryptobacteroides sp900544195**	0.365	0.00009	<0.0001
*Bacteroidota/Bacteroidia/Bacteroidales/UBA932/***Cryptobacteroides sp900317925**	0.209	0.00003	<0.0001
*Bacteroidota/Bacteroidia/Bacteroidales/UBA932/***Cryptobacteroides sp902785575**	0.235	0.00002	<0.0001
*Bacteroidota/Bacteroidia/Bacteroidales/UBA932/***Cryptobacteroides sp900316045**	0.102	0.00004	<0.0001
**Cluster 13**	**0.175**	**0.058**	**0.003**
*Bacillota/Clostridia/Oscillospirales/Acutalibacteraceae/***UBA5905 sp002437905**	0.089	0.00005	<0.0001
*Bacillota/Clostridia/Christensenellales/CAG-138/***SFEL01 sp004557245**	0.155	0.00006	<0.0001
*Bacillota/Clostridia/Christensenellales/CAG-138/***PeH17 sp000435055**	0.190	0.00008	<0.0001
*Bacillota/Bacilli/RF39/UBA660/***CAG-533 sp000434495**	0.186	0.00006	<0.0001
*Bacillota/Bacilli/RF39/UBA660/***CAG-302 sp000431795**	0.245	0.00009	<0.0001
*Bacillota/Bacilli/RF39/UBA660/***CAG-914 sp000437895**	0.145	0.00007	<0.0001
*Bacillota/Bacilli/RF39/UBA660/***CAG-710 sp000432595**	0.236	0.00007	<0.0001
*Bacillota/Bacilli/RF39/UBA660/***UMGS872 sp900546275**	0.149	0.00007	<0.0001
*Bacillota/Bacilli/RF39/UBA660/***Onthocola_B sp000437355**	0.276	0.00009	<0.0001

*Bacillota/Bacilli/RF39/UBA660/***CAG-628 sp003524085**	0.106	0.00005	<0.0001
*Bacillota/Bacilli/RF39/UBA660/***RUG705 sp900551455**	0.091	0.00006	<0.0001
*Bacillota/Clostridia/TANB77/CAG-508/***UMGS1994 sp900553945**	0.223	0.00006	<0.0001

[a]Effect size refers to log-fold change in the differential abundance analyses and β coefficient in the cluster analyses.
[b]Associations between the CRC lifestyle index and species level taxa were assessed using Analysis of Compositions of Microbiomes with Bias Correction 2 (ANCOM-BC2). Species that were statistically significantly associated with the CRC lifestyle index, and passed the internal robustness screening in ANCOM-BC2, were included in the cluster analysis. The analyses were adjusted for sex, age, energy intake (MJ/d), smoking habits (smoker/nonsmoker), and use of potentially microbiome altering medication (yes/no; metformin, psycholeptics, psychoanaleptics, proton pump inhibitors and constipation medication).
